# Effect of Commercially Available Sugar-Sweetened Beverages on Subjective Appetite and Short-Term Food Intake in Boys

**DOI:** 10.3390/nu11020270

**Published:** 2019-01-26

**Authors:** Kelly L. Poirier, Julia O. Totosy de Zepetnek, Lorianne J. Bennett, Neil R. Brett, Terence Boateng, Alexander Schwartz, Bohdan L. Luhovyy, Nick Bellissimo

**Affiliations:** 1 Department of Applied Human Nutrition, Mount Saint Vincent University, 166 Bedford Highway, Halifax, NS B3M 2J6, Canada; poirierkelly@hotmail.com (K.L.P.); loriannebennett@gmail.com (L.J.B.); Bohdan.luhovyy@msvu.ca (B.L.L.); 2 Faculty of Kinesiology & Health Studies, University of Regina, 3737 Wascana Parkway, Regina, SK S4S 0A2, Canada; julia.totosy@uregina.ca; 3 School of Nutrition, Ryerson University, 350 Victoria Street, Toronto, ON M5B 2K3, Canada; neil.brett@ryerson.ca (N.R.B.); tboateng@ryerson.ca (T.B.); alexander.schwartz@ryerson.ca (A.S.)

**Keywords:** food intake, appetite, chocolate milk, fruit drink, cola, children

## Abstract

It is unclear whether sugar sweetened beverages bypass regulatory controls of food intake (FI) in boys. The objective of the present study was to determine the effects of isovolumetric preloads (350 mL) of a fruit-flavoured drink (154 kcal), cola (158 kcal), 1% M.F. chocolate milk (224 kcal), and water (0 kcal) on subjective appetite and FI in boys aged 9–14 years. On four separate mornings, boys consumed one of the preloads in a random order; subjective appetite was measured at 15 min intervals, and FI was measured via an ad libitum pizza lunch at 60 min post-beverage consumption. In the 32 boys (age: 11.8 ± 0.3 years), FI was reduced (*p* < 0.001) after cola (940 ± 46 kcal) and chocolate milk (878 ± 41 kcal) compared with the water control (1048 ± 35 kcal) and after chocolate milk compared to the fruit drink (1005 ± 44 kcal). Cumulative FI after the fruit drink was greater than the water control (1159 ± 44 vs. 1048 ± 35 kcal; *p* = 0.03). Average appetite was not affected by the treatment, but the cola treatment resulted in greater fullness (*p* = 0.04) and lower prospective food consumption (*p* = 0.004) compared with the fruit drink. In conclusion, chocolate milk and cola suppressed next-meal FI at 60 min, while fruit drink increased cumulative FI (beverage + next meal) over 60 min in boys. Results from this study suggest that beverage composition is an important determinant of FI suppression in boys.

## 1. Introduction

Beverages account for up to 20% of daily caloric intake in Canadian children aged 4**–**18 y, and 44% of the average daily sugars intake of children is derived from fluid dairy (14%), fruit juices (9%), soft drinks (14%), and fruit drinks (7%) [1,2]. It has been hypothesized that sugars-sweetened beverages bypass the regulatory control mechanisms of food intake (FI), leading to overconsumption of food and subsequent weight gain, which has prompted the development of beverage recommendations to limit their consumption [3]. It is important to understand factors contributing to unhealthy body weights in children, as the prevalence of childhood obesity in Canada has doubled over the past 30 years and one in three children are either overweight or obese [4].

It is unclear whether the sugars source, composition, or form are primary determinants of FI control in children. We previously reported that the effect of glucose and whey-protein solutions on short-term FI in healthy-weight boys is both source and time dependent [5]. Energy compensation was diminished following the consumption of 50 g glucose in solution when the delay interval between the beverage consumption and test meal was extended from 30 to 60 min, whereas the effect of whey-protein was increased [5]. Despite these observations suggesting that proteins exert a stronger effect than sugars on FI suppression in healthy-weight boys, there are limited reports of familiar, commercially available sugar-sweetened beverages on subjective appetite and FI in boys. 

Work in healthy young adults [6] and children 9–14 years [7] showed that cumulative energy intake was higher after the consumption of caloric beverages compared to water. However, in girls 9–14 years, the consumption of 350 mL of chocolate milk, cola, or a fruit drink 60 min before a meal did not significantly increase the cumulative energy intake compared with a water control [8]. These results may be due to observed sex differences in FI regulation, with males consuming more energy than females even after adjusting for fat mass, fat-free mass, height, and weight statuses [9]. Combined with work demonstrating that girls have more restrained eating behaviors [10], energy compensation from caloric beverages may indeed be affected by sex differences. It is unclear whether cumulative energy intake is affected by pre-meal consumption of sugar-sweetened beverages in boys. Therefore, the objective of the present study was to determine the effect of isovolumetric preloads of commercially available sugar-sweetened beverages, varying in macronutrient composition, on subjective appetite and FI in boys aged 9–14 years.

## 2. Materials and Methods

### 2.1. Participants

Boys between the ages of 9 and 14 years were recruited to participate in this study. Body Mass Index (BMI) percentile for age and sex was defined using the US Centers for Disease Control and Prevention growth charts [11]. Boys were recruited primarily by word-of-mouth; through flyers around Halifax Regional Municipality where the parents of the school-aged children frequented, such as libraries and community centers; and by advertisements in the local newspaper. Baseline characteristics of the participants are reported in Table 1. The Mount Saint Vincent University’s Research Ethics Board approved the study. 

Boys who were born full-term and had a normal birth weight were included. Individuals dieting or taking medication and those with any significant learning, behavioral, or emotional difficulties were excluded. If a child met the study requirements, an appointment was made for the child and parent at the Department of Applied Human Nutrition for a screening session. During this session, informed consent and assent were obtained from the parent and child respectively, anthropometrics and body composition were measured, and pizza preference was recorded. The parent and child were given a tour of the facility in order to familiarize the child with the protocols and minimize apprehension during the first test visit, as previously reported [5,12,13,14,15]. 

### 2.2. Experimental Design

This randomized trial was a within-subjects repeated measures design. Following the familiarization session, participants came to the laboratory for 4 visits. On each visit, participants arrived at the Department of Applied Human Nutrition at 10:00 or 11:00 am (consistent over the 4 visits), 2 h after consuming a standardized breakfast at home. The standardized breakfast consisted of fat-free skim milk (250 mL, 90 kcal; Baxter, Saint John, NB, Canada), breakfast cereal (26 g, 102 kcal; Honey Nut Cheerios®, General Mills, Mississauga, ON, Canada), and Tropicana® orange juice (236 mL, 110 kcal; Tropicana Products Inc, Bradenton, FL, USA). Upon arrival, a research assistant verified that the breakfast was consumed with both the child and parent. If the child did not consume their entire breakfast, the session was rescheduled. Children then completed a motivation-to-eat visual analogue scale (VAS) questionnaire measuring their subjective appetite, as reported in our previous publications [5,8,12,13,14,15,16]. Boys then received, in a random order, one of the four isovolumetric (350 mL) test preloads in chilled opaque cups: fruit drink (154 kcal; Fruite®, A. Lassonde Inc., Montreal, QC, Canada), cola (158 kcal; Coca Cola®, Toronto, ON, Canada), chocolate milk (224 kcal; Baxter’s® 1% M.F. Chocolate Milk, Saint John, NB, Canada) or a water control (0 kcal; Nestle Pure Life®, Toronto, ON, Canada). The boys were instructed to consume the preload within 5 min; sweetness and pleasantness of the preloads were measured using a VAS. The nutritional compositions of the preloads are reported in our previous study [8]. 

Sixty minutes following beverage consumption, the boys were escorted to a taste panel room, seated in individual cubicles free of most external cues, and were served an *ad libitum* pizza lunch along with a 500 mL bottle of water. The participants were informed that additional hot trays of pizza would be provided at regular intervals and were asked to eat until they were comfortably full. Subjective appetite via VAS was assessed at 15 min intervals post-beverage consumption, as well as following the *ad libitum* pizza meal. 

### 2.3. Experimental Procedures

#### 2.3.1. Anthropometrics and Body Composition

Anthropometric measurements of height (m) and body mass (kg) were measured, and body mass index (BMI, kg/m^2^ and percentile) was calculated using the US Centers for Disease Control and Prevention growth charts [11]. Skinfold thickness recorded to the nearest 0.1 mm at the triceps, biceps, suprailiac, and subscapular sites were obtained by standard procedures using a Lange skinfold caliper (Cambridge Scientific Industries, Cambridge, MD, USA) [17]. The mean of three consecutive skinfold measurements was used for statistical analyses. 

#### 2.3.2. Food Intake

Two varieties of Deep ‘N Delicious pizza (pepperoni and three cheese, donated by McCain Canada Ltd., Florenceville, NB, Canada) were served for lunch based on the boys’ preference, as previously reported [8,18,19]. Three pizzas were cooked, weighed, and cut into four equal pieces before serving in 10 min intervals, and FI was measured by subtracting the amount of pizza left after the meal from the initial weight. Nutritional composition provided by the manufacturer was used to convert the net weight consumed to kilocalories. The bottled water was also weighed before and after the test meal to calculate the net amount ingested during the meal. 

#### 2.3.3. Subjective Appetite and Thirst

VASs were used to assess subjective appetite and the sweetness and pleasantness of the beverages and pizza, as described in our previous publication [8]. Briefly, subjective appetite was measured using the following four questions: (1) desire to eat, (2) hunger, (3) fullness, and (4) prospective food consumption (PFC), and an average appetite score was calculated using the formula ((desire to eat) + (hunger) + (100 − fullness) + (PFC)/4). This subjective appetite VAS was previously validated in children [13]. VAS for subjective appetite was assessed at baseline (0 min) and at 15, 30, 45, and 60 min post-beverage consumption. VAS related to sweetness and pleasantness of the beverage and pizza were completed immediately after beverage consumption and the test meal, respectively. Subjective thirst was assessed using a VAS at 0, 15, 30, 45 and 60 min with the following anchors: “not thirsty at all” to “as thirsty as I ever felt”.

### 2.4. Statistical Analysis

Statistical Analysis Systems version 9.2 (SAS Institute Inc., Carey, NC, USA) was used to perform all statistical analyses. Data are reported as mean ± SEM (standard error of the mean). The effects of the beverage treatment (fruit drink, cola, chocolate milk, of water) on FI, water intake, caloric compensation (CC), sweetness and pleasantness of the preloads, and pleasantness of the test meal were analyzed using a one-factor repeated measures ANOVA. When main effects were found, post-hoc analyses were completed using Tukey**–**Kramer’s test, adjusted for multiple comparisons. A two-factor repeated measures ANOVA was used to determine the effect of preload and time on the change from the baseline subjective average appetite and subjective thirst. Pearson correlation coefficients were calculated to assess associations between dependent measures.

As previously reported [5,8,12,16,20], CC following each preload was calculated by the following formula:

CC(%) = (FI after control (kcal) − FI after the preload (kcal))/(kcal in the preload) × 100(1)

Compensation scores of less than 100% indicated failure to fully compensate at the test meal, whereas scores above 100% indicated that the boys overcompensated by consuming less energy at the test meal than that contained in the preload.

## 3. Results

Thirty-two boys (age: 11.8 ± 0.3 years; body mass: 50.3 ± 2.5 kg; BMI percentile: 72.2 ± 5.3%) completed the study. The baseline characteristics are summarized in Table 1.

FI was reduced after chocolate milk (16%, *p* < 0.001) and cola (10%, *p* = 0.02) compared to the water control, but there were no differences in FI between these two treatments (*p* = 0.33) (Table 2). FI was higher after the fruit drink compared to chocolate milk (13%, *p* = 0.01), and consuming the fruit drink resulted in a 10% increase in cumulative FI (kcal preload + kcal test meal) compared with the water control (*p* = 0.02; Table 2). Neither water intake (*p* = 0.09) nor CC (*p* = 0.11) differed among the test beverages.

### Subjective Ratings from Visual Analog Scales

**Figure 1:**
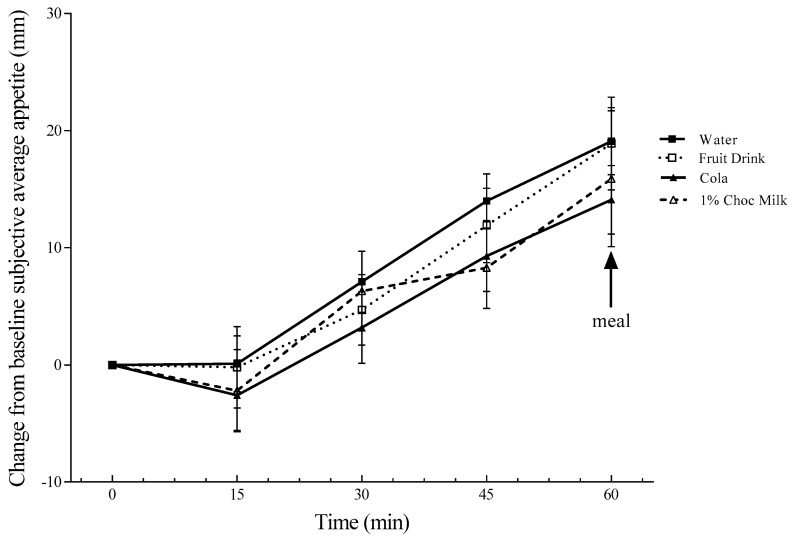
The change from the baseline subjective average appetite over 60 min. The values are mean ± SEM (*n* = 32). The change from the baseline subjective average appetite was affected by time (*p* < 0.001).

**Figure 2:**
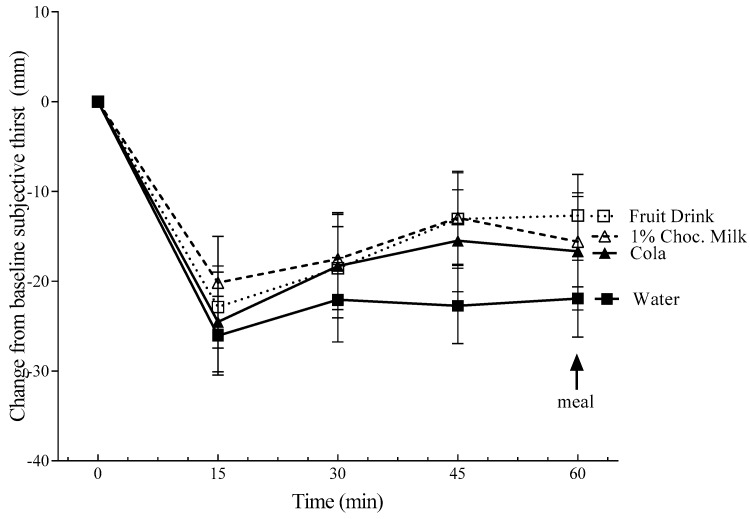
The change from the baseline subjective thirst over 60 min. The values are mean ± SEM (*n* = 32). The change from the baseline scores was affected by the treatment (*p* = 0.027) and time (*p* = 0.014). Subjective thirst was significantly lower after water compared with the fruit drink (*p* = 0.048) and 1% chocolate milk (*p* = 0.037).

The change from baseline subjective average appetite was not affected by the preload but increased over time (*p* < 0.001) (Figure 1). The change from baseline subjective thirst scores were affected by the treatment (*p* = 0.027) and time (*p* = 0.014) such that thirst was lower after water compared to fruit drink (*p* = 0.048) and 1% chocolate milk (*p* = 0.037) but not cola (Figure 2). Preload sweetness (*p* = 0.02) and pleasantness (*p* = 0.006) were affected by the preload treatments such that the fruit drink was rated sweeter than cola (*p* = 0.032) and chocolate milk (*p* = 0.036; Table 2). Additionally, chocolate milk was rated more pleasant than cola (*p* = 0.006). Neither the subjective mean sweetness (*r* = 0.26, *p* = 0.15) nor pleasantness (*r* = 0.07, *p* = 0.49) correlated with the mean FI. 

## 4. Discussion

The present study investigated the effects of consuming three different commercially available sugars-sweetened beverages compared to water on subjective appetite and next-meal FI in boys. The main findings suggest that beverage type is a determinant of short-term FI suppression in boys. Although the subjective average appetite was not affected by beverage type, the next meal FI was lower following the consumption of chocolate milk compared with water and fruit drinks. In addition, cumulative FI was greater after the fruit drink compared with water. 

Similar to results in 9–14 years old girls [8], the fruit drink failed to suppress test meal FI compared to water. It is possible that the fruit drink failed to suppress FI due to its glucose to fructose ratio, where fructose has been shown to have a weaker effect on satiety hormone release [22]. Although cola has similar glucose and energy content to the fruit drink and its fructose content is actually higher, it is possible cola suppressed FI due to its effect on gastric distension related to carbon dioxide release as previously described [8]. Further work in this area in boys is needed as the effect of carbonation on FI regulation in adults may last less than 15 min [23], but studies in boys are lacking. 

Cumulative FI was greater after consuming the fruit drink when compared with water; these findings are contrary to our previous report in girls [8]. This effect could be explained by sex differences regarding appetite hormones such as leptin prior to and during puberty. Leptin is responsible for energy homeostasis through its effects on stimulating satiety [24] and is necessary for normal pubertal development. Leptin increases prior to pubertal onset and stimulates the release of gonadotropins necessary for pubertal development [25]. Unlike girls, leptin in boys rapidly decreases at the onset of puberty and could, therefore, result in greater FI through decreased satiety signals [26]. However, we did not measure plasma leptin or assess pubertal stage in this study. 

Consumption of the fruit drink resulted in less FI suppression compared to chocolate milk and a greater cumulative FI compared to water. It is unlikely FI was influenced by the kilocalorie difference between chocolate milk and the fruit drink (224 kcal vs. 154 kcal), as previous studies in young adults showed that between-beverage energy content differences of 30 to 170 kcal did not affect FI 30 min or 120 min after beverage consumption [27]. A possible explanation for the FI suppressing effects of chocolate milk vs. fruit drink is the protein [28] and sucrose [29] content of the beverages. Protein suppression of FI may result from bioactive peptides and amino acids released after their digestion, as well as whey and casein proteins both potentially increasing satiety hormones (e.g. cholecystokinin (CCK)) [30]. Though little work examining protein doses similar to our current study exists for boys, in healthy adults, whey protein (10–20 g) suppressed FI at 30 min [28] but not 120 min [31]. Lastly, although glucose has been shown to suppress FI in boys 9–14 years [19], there are mixed results as to the effect of sucrose on FI regulation [19,29,32], and research is lacking regarding the effect of lactose on FI regulation in children and adolescents. 

The subjective average appetite and subjective average appetite per kilocalorie were not affected by the treatments. Further, similar to previous results in boys [5], appetite did not decrease after consuming the test beverages (Figure 1). This may be explained, in part, by beverages being less satiating than solid foods in children [33]. However, it has also been hypothesized that weight status affects appetite [34,35]. Overweight and obese boys were pooled with normal weight boys in the present study, as the effect of each beverage on appetite and FI did not differ by weight status. This may be a consequence of the fixed beverage size for all participants rather than providing a quantity that was relative to body weight. In previous experiments, it has been shown that after a fixed glucose load, FI was decreased similarly in normal weight and overweight/obese boys, but FI was only reduced in normal weight boys after a fixed whey protein load. However, whey-protein suppression of FI was greater than glucose when it was provided relative to body weight in healthy-weight boys [5]. 

Subjective sweetness and pleasantness ratings differed among the preloads; however, it is unlikely these factors were primary determinants of FI. It has been suggested that sweetness may increase appetite due to the stimulation of gut taste receptors [36]. Importantly however, although the fruit drink was rated significantly sweeter than cola, test meal and cumulative FI did not differ between these beverages. Also, it was recently shown in children that the difference in sweetness between chocolate milk and a fruit drink did not affect FI at a subsequent meal [7]. These findings suggest that either the magnitude of difference in sweetness between cola and the fruit drink was not sufficient to affect FI or that sweetness is not a strong determinant of FI in boys, as previously reported [5]. FI was similar after cola and chocolate milk in our study, and chocolate milk was rated more pleasant than cola, suggesting pleasantness was not an important determinant of FI. Likewise, previous research in adults [37] and children [7,19] have reported preload pleasantness was not strongly associated with FI. 

Although the design of this study provides valuable information about the relationship between sugar-containing beverages and subsequent FI in boys, there were several limitations. The effect of carbonation could not be investigated, as uncarbonated cola was not included in the study. By design, this trial investigated the between-meal effect of beverage consumption on FI and satiety, meaning that further work is needed to understand if within-meal beverage consumption has a similar effect on FI, as suggested from emerging data in children [7]. Further, it is unknown if short-term FI in children would translate into changes in body weight, which would require a longitudinal study design to confirm our findings. Lastly, this study did not investigate physiological mechanisms associated with FI, as done in some previous work in children [7,34]. 

## 5. Conclusions

Chocolate milk and cola suppressed FI at 60 min, while fruit drink increased cumulative FI at 60 min in boys. Results from this study suggest that beverage composition is an important determinant of FI suppression in boys.

## Figures and Tables

**Table 1:** The baseline characteristics of the participants.

Participant Characteristics (*n* = 32)	Mean ± SEM ^1^
Age (year)	11.8 ± 0.3
Body Mass (kg)	50.3 ± 2.5
Height (m)	1.5 ± 0.02
BMI Percentile	72.2 ± 5.3
Fat Mass (kg)	10.1 ± 1.3
Fat Mass (%)	19.4 ± 1.9
Fat-Free Mass (kg)	40.1 ± 2.0
Fat-Free Mass (%)	80.6 ± 1.9

^1^ SEM = standard error of the mean; BMI = body mass index. Fat and fat-free mass were estimated from the sum of the skinfold measurements at four points [21].

**Table 2:** The effect of preload beverages on food and water intake at 60 min and on the sweetness and pleasantness of the beverages and pizza ^1^.

	Water	Fruit Drink	Cola	Chocolate Milk	*p*-Value
Food Intake (kcal)	1048 ± 35 ^a^	1005 ± 44 ^a,b^	940 ± 46 ^b,c^	878 ± 41 ^c^	<0.0001
Cumulative Food Intake (kcal)	1048 ± 35 ^a^	1159 ± 44 ^b^	1098 ± 46 ^a,b^	1102 ± 41 ^a^^,b^	0.03
Caloric Compensation (%)	-	30 ± 24	68 ± 25	76 ± 12	0.11
Water Intake (g)	200 ± 33	249 ± 34	240 ± 32	250 ± 29	0.09
Preload Sweetness (mm)	-	80 ± 4 ^a^	68 ± 5 ^b^	68 ± 4 ^b^	0.02
Preload Pleasantness (mm)	-	73 ± 5 ^a,b^	66 ± 6 ^a^	86 ± 3 ^b^	0.006
Test Meal Pleasantness (mm)	87 ± 3	89 ± 3	86 ± 4	81 ± 4	0.08

^1^ Data are presented as means ± SEM; *n* = 32. ^a–c^ Different superscripts within a row show significant differences (*p* < 0.05) using a one-factor mixed model ANOVA with a Tukey–Kramer post-hoc correction.
